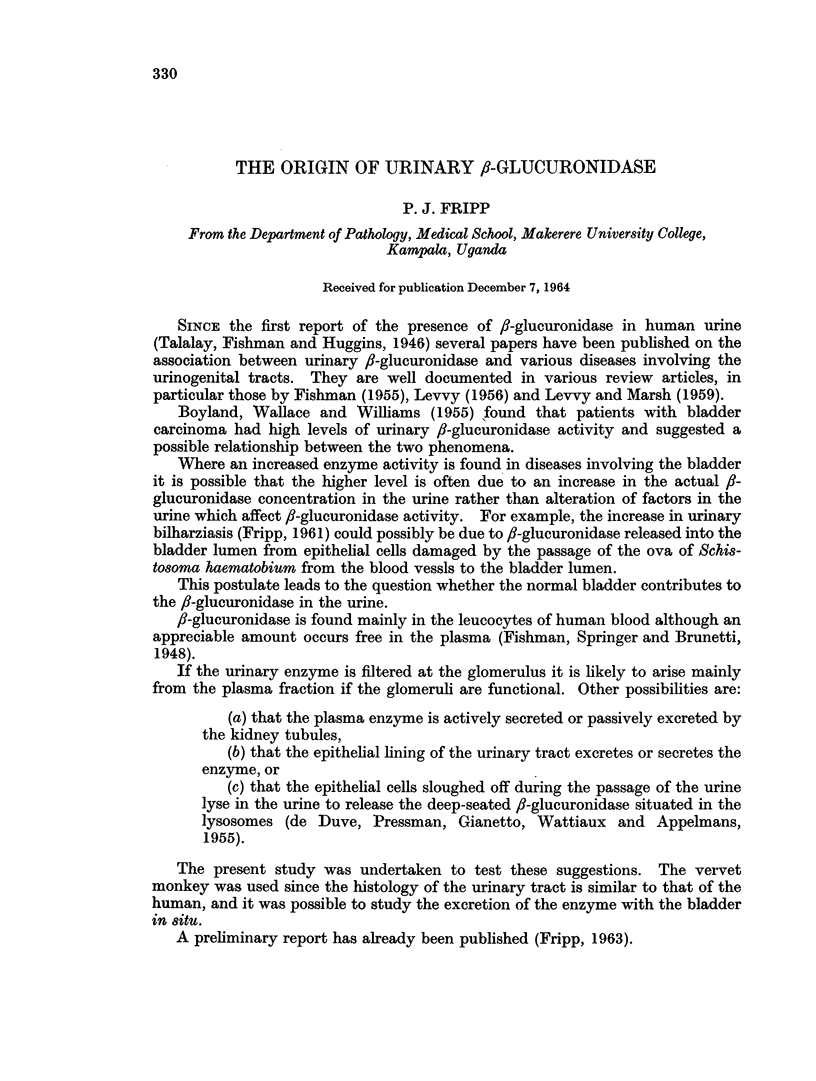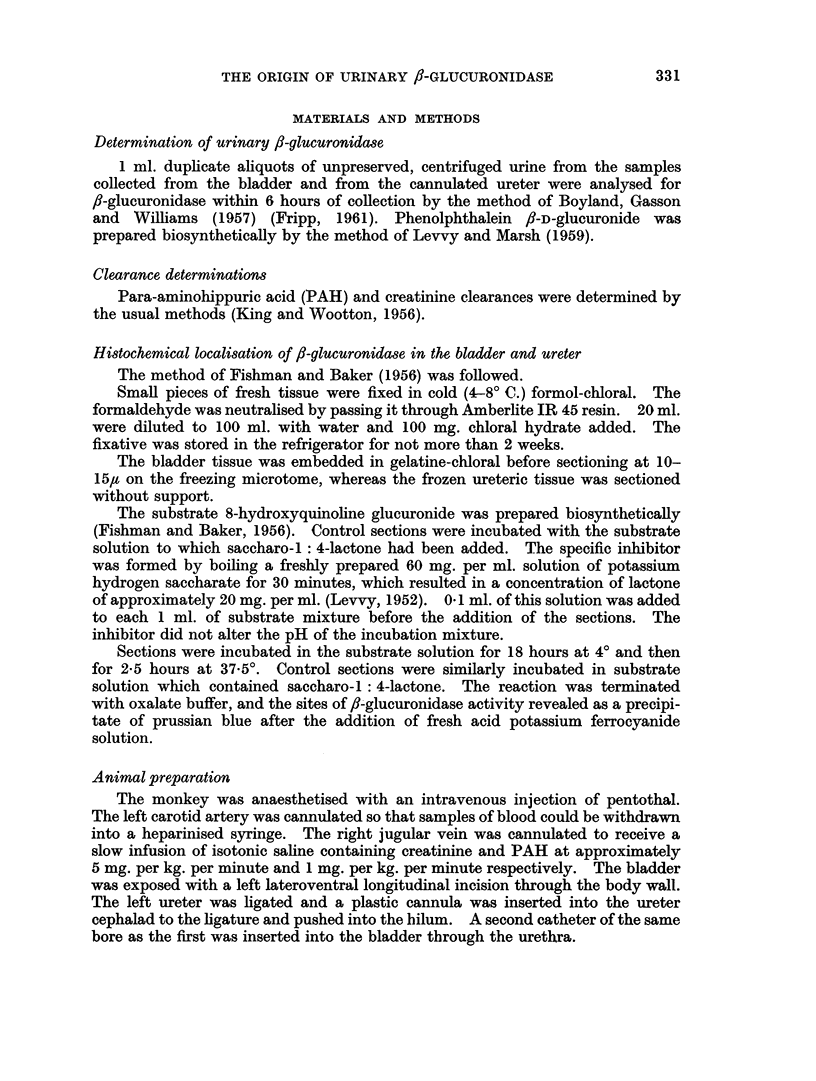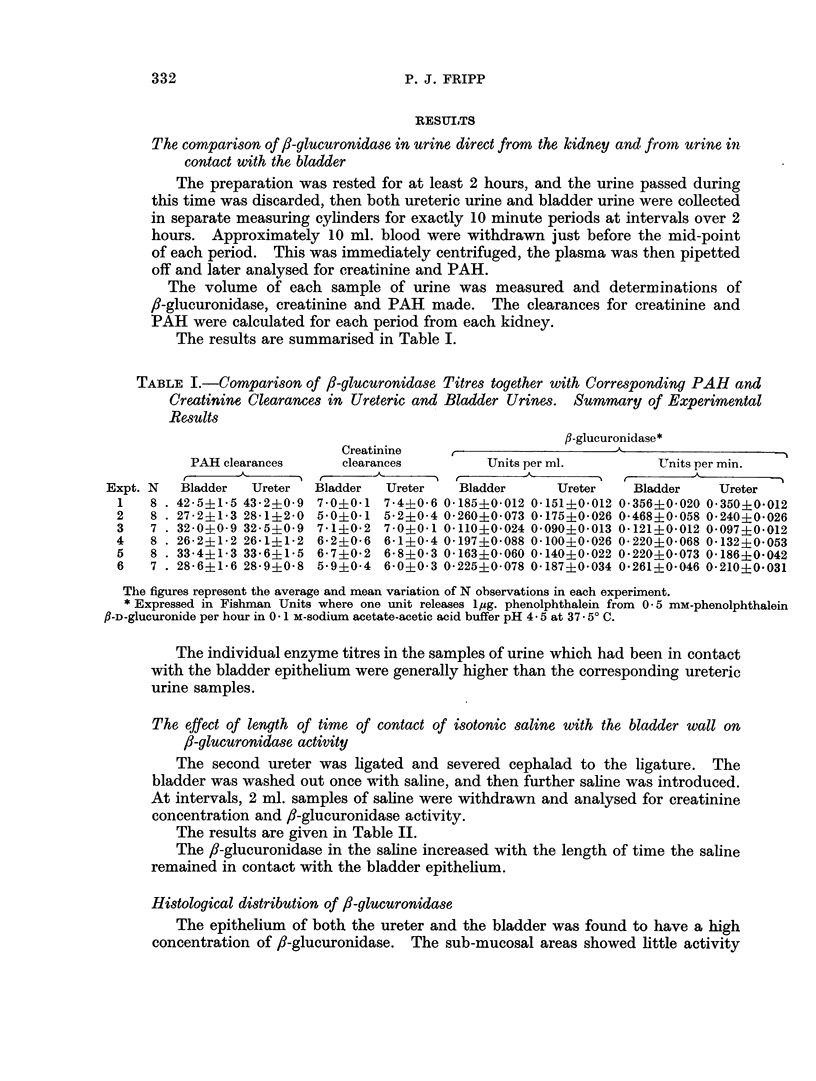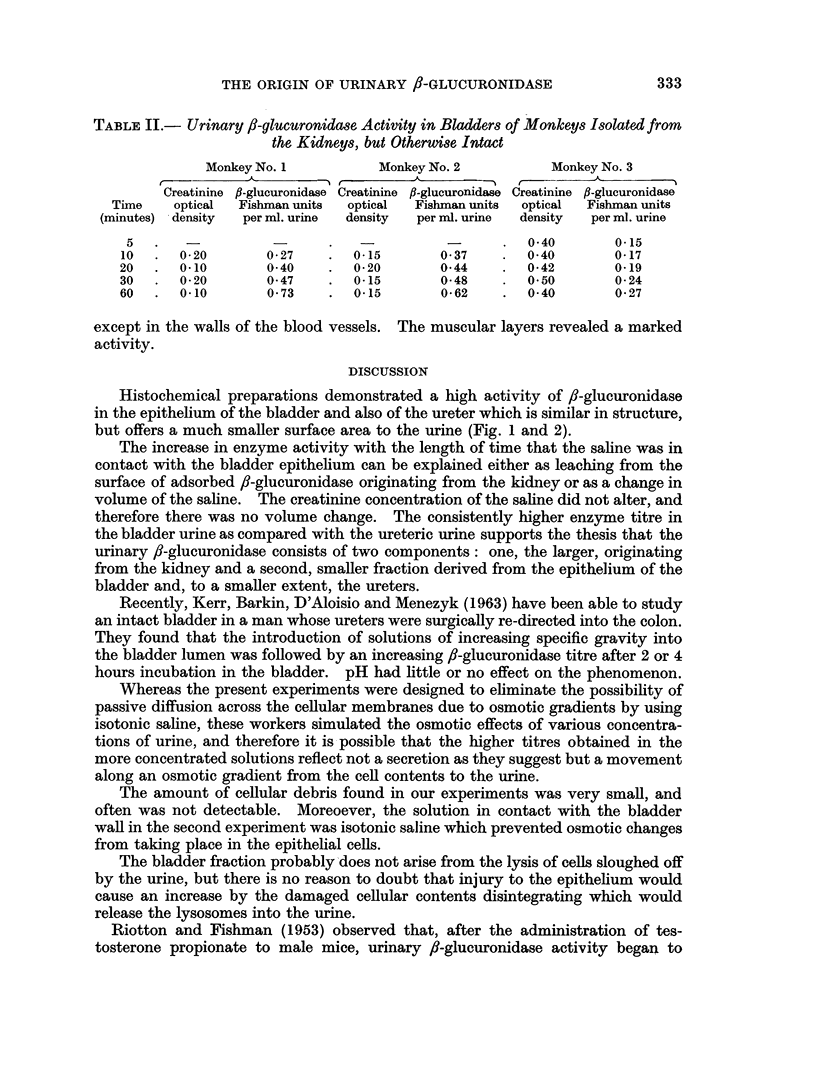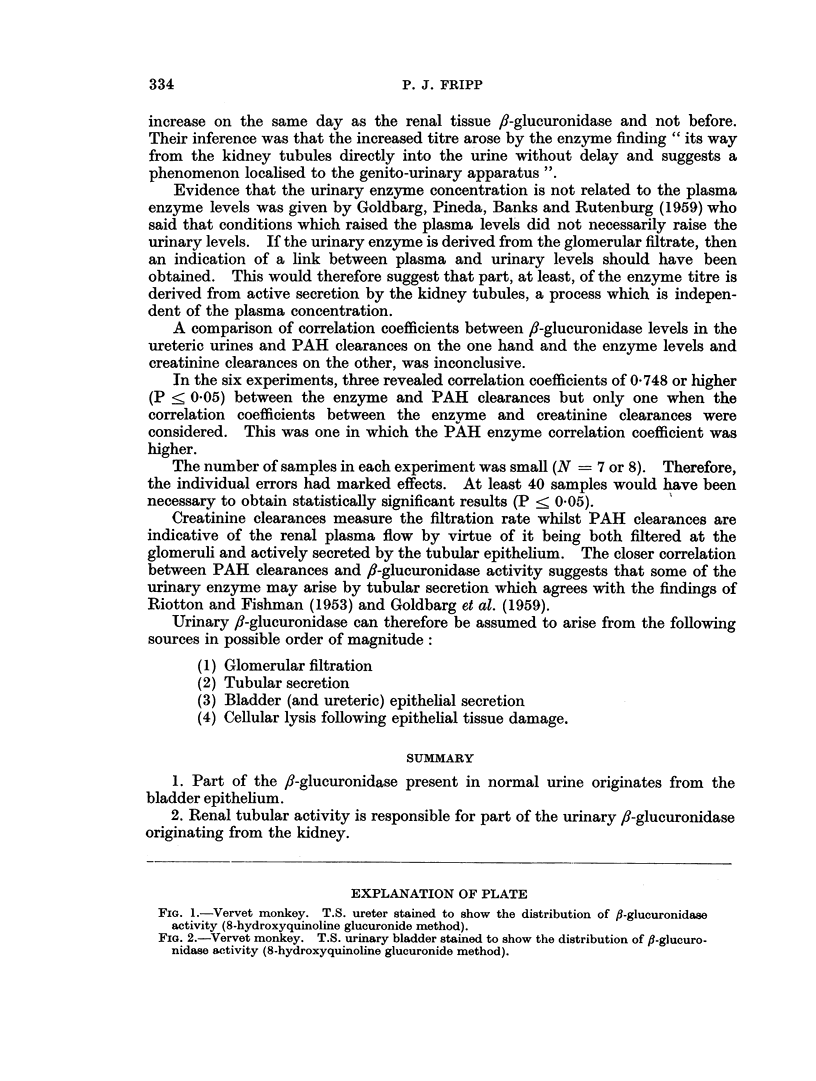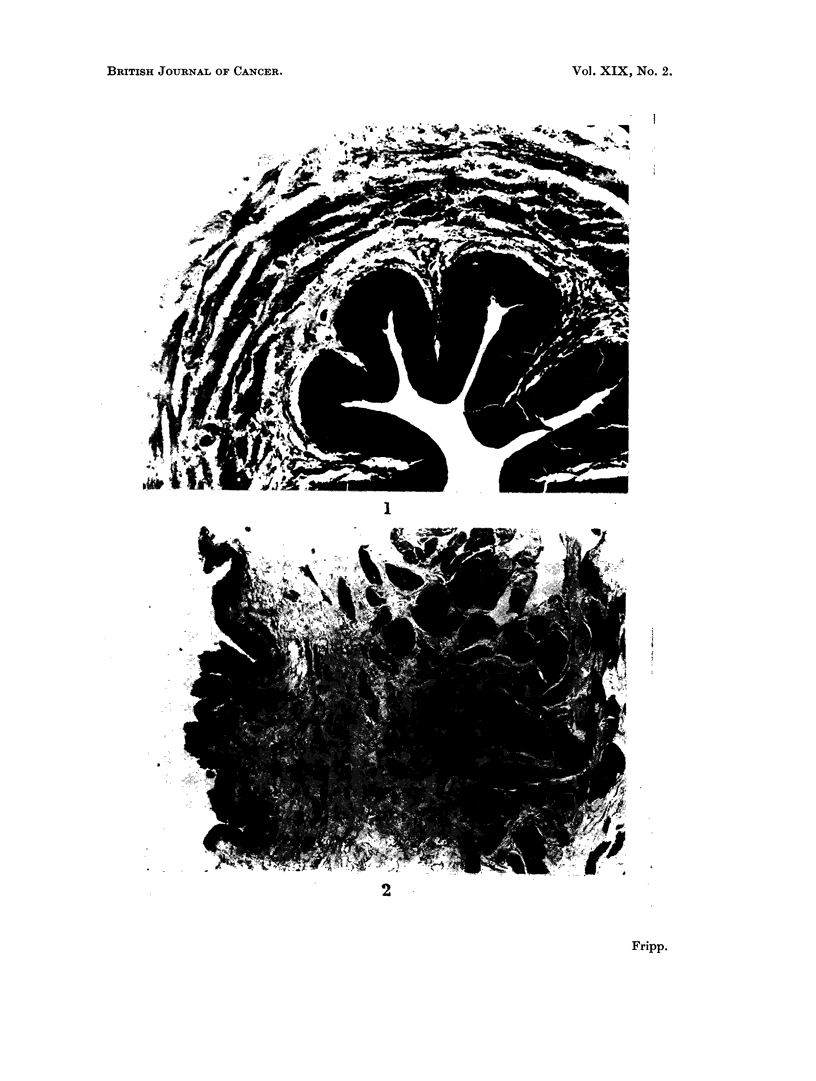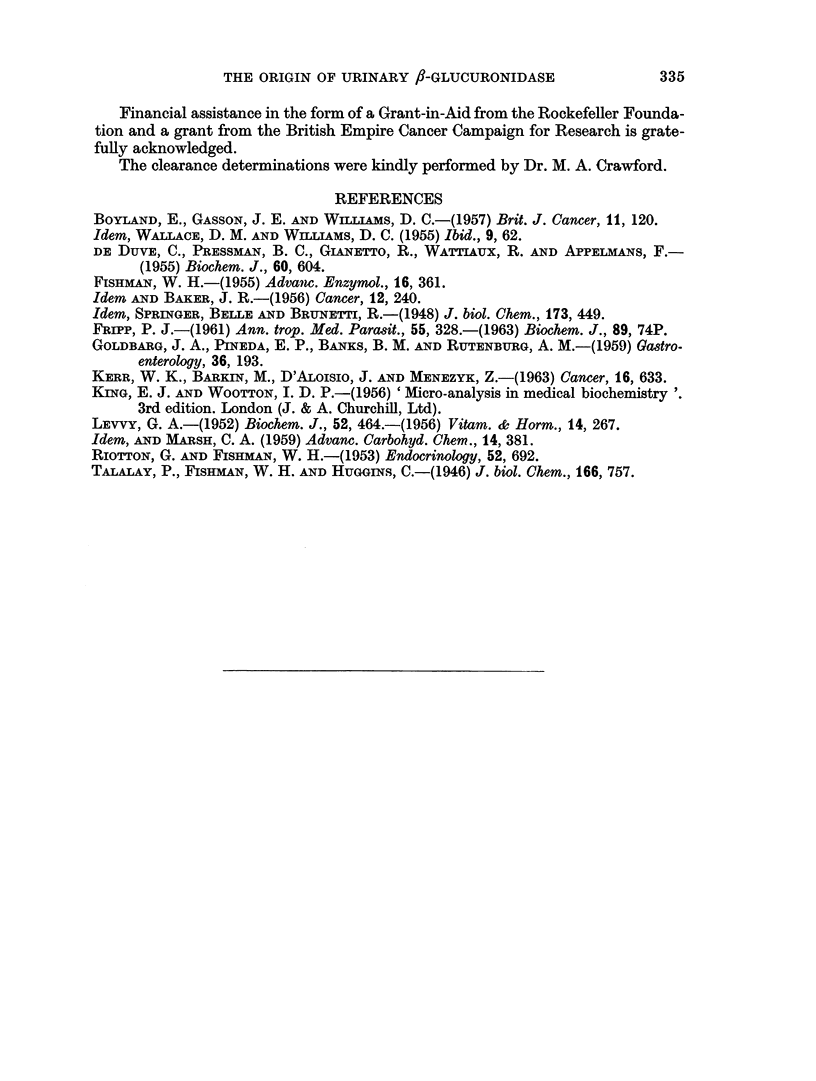# The Origin of Urinary β-Glucuronidase

**DOI:** 10.1038/bjc.1965.38

**Published:** 1965-06

**Authors:** P. J. Fripp

## Abstract

**Images:**


### THE ORIGIN OF URINARY β-GLUCURONIDASE

P. J. FRIPP

*From the Department of Pathology, Medical School, Makerere University College, Kampala, Uganda*

Received for publication December 7, 1964

SINCE the first report of the presence of β-glucuronidase in human urine (Talalay, Fishman and Huggins, 1946) several papers have been published on the association between urinary β-glucuronidase and various diseases involving the urinogenital tracts. They are well documented in various review articles, in particular those by Fishman (1955), Levvy (1956) and Levvy and Marsh (1959).

Boyland, Wallace and Williams (1955) found that patients with bladder carcinoma had high levels of urinary β-glucuronidase activity and suggested a possible relationship between the two phenomena.

Where an increased enzyme activity is found in diseases involving the bladder it is possible that the higher level is often due to an increase in the actual β-glucuronidase concentration in the urine rather than alteration of factors in the urine which affect β-glucuronidase activity. For example, the increase in urinary bilharziasis (Fripp, 1961) could possibly be due to β-glucuronidase released into the bladder lumen from epithelial cells damaged by the passage of the ova of *Schistosoma haematobium* from the blood vessls to the bladder lumen.

This postulate leads to the question whether the normal bladder contributes to the β-glucuronidase in the urine.

β-glucuronidase is found mainly in the leucocytes of human blood although an appreciable amount occurs free in the plasma (Fishman, Springer and Brunetti, 1948).

If the urinary enzyme is filtered at the glomerulus it is likely to arise mainly from the plasma fraction if the glomeruli are functional. Other possibilities are:

(*a*) that the plasma enzyme is actively secreted or passively excreted by the kidney tubules,

(*b*) that the epithelial lining of the urinary tract excretes or secretes the enzyme, or

(*c*) that the epithelial cells sloughed off during the passage of the urine lyse in the urine to release the deep-seated β-glucuronidase situated in the lysosomes (de Duve, Pressman, Gianetto, Wattiaux and Appelmans, 1955).

The present study was undertaken to test these suggestions. The vervet monkey was used since the histology of the urinary tract is similar to that of the human, and it was possible to study the excretion of the enzyme with the bladder *in situ*.

A preliminary report has already been published (Fripp, 1963).

### MATERIALS AND METHODS

*Determination of urinary β-glucuronidase*

1 ml. duplicate aliquots of unpreserved, centrifuged urine from the samples collected from the bladder and from the cannulated ureter were analysed for β-glucuronidase within 6 hours of collection by the method of Boyland, Gasson and Williams (1957) (Fripp, 1961). Phenolphthalein β-D-glucuronide was prepared biosynthetically by the method of Levvy and Marsh (1959).

*Clearance determinations*

Para-aminohippuric acid (PAH) and creatinine clearances were determined by the usual methods (King and Wootton, 1956).

*Histochemical localisation of β-glucuronidase in the bladder and ureter*

The method of Fishman and Baker (1956) was followed.

Small pieces of fresh tissue were fixed in cold (4–8° C.) formol-chloral. The formaldehyde was neutralised by passing it through Amberlite IR 45 resin. 20 ml. were diluted to 100 ml. with water and 100 mg. chloral hydrate added. The fixative was stored in the refrigerator for not more than 2 weeks.

The bladder tissue was embedded in gelatine-chloral before sectioning at 10–15$\mu$ on the freezing microtome, whereas the frozen ureteric tissue was sectioned without support.

The substrate 8-hydroxyquinoline glucuronide was prepared biosynthetically (Fishman and Baker, 1956). Control sections were incubated with the substrate solution to which saccharo-1 : 4-lactone had been added. The specific inhibitor was formed by boiling a freshly prepared 60 mg. per ml. solution of potassium hydrogen saccharate for 30 minutes, which resulted in a concentration of lactone of approximately 20 mg. per ml. (Levvy, 1952). 0·1 ml. of this solution was added to each 1 ml. of substrate mixture before the addition of the sections. The inhibitor did not alter the pH of the incubation mixture.

Sections were incubated in the substrate solution for 18 hours at 4° and then for 2·5 hours at 37·5°. Control sections were similarly incubated in substrate solution which contained saccharo-1 : 4-lactone. The reaction was terminated with oxalate buffer, and the sites of β-glucuronidase activity revealed as a precipitate of prussian blue after the addition of fresh acid potassium ferrocyanide solution.

*Animal preparation*

The monkey was anaesthetised with an intravenous injection of pentothal. The left carotid artery was cannulated so that samples of blood could be withdrawn into a heparinised syringe. The right jugular vein was cannulated to receive a slow infusion of isotonic saline containing creatinine and PAH at approximately 5 mg. per kg. per minute and 1 mg. per kg. per minute respectively. The bladder was exposed with a left lateroventral longitudinal incision through the body wall. The left ureter was ligated and a plastic cannula was inserted into the ureter cephalad to the ligature and pushed into the hilum. A second catheter of the same bore as the first was inserted into the bladder through the urethra.

### RESULTS

*The comparison of β-glucuronidase in urine direct from the kidney and from urine in contact with the bladder*

The preparation was rested for at least 2 hours, and the urine passed during this time was discarded, then both ureteric urine and bladder urine were collected in separate measuring cylinders for exactly 10 minute periods at intervals over 2 hours. Approximately 10 ml. blood were withdrawn just before the mid-point of each period. This was immediately centrifuged, the plasma was then pipetted off and later analysed for creatinine and PAH.

The volume of each sample of urine was measured and determinations of β-glucuronidase, creatinine and PAH made. The clearances for creatinine and PAH were calculated for each period from each kidney.

The results are summarised in Table I.

TABLE I.—*Comparison of β-glucuronidase Titres together with Corresponding PAH and Creatinine Clearances in Ureteric and Bladder Urines. Summary of Experimental Results*

| | | PAH clearances | | Creatinine clearances | | β-glucuronidase* Units per ml. | | β-glucuronidase* Units per min. | |
|---|---|---|---|---|---|---|---|---|---|
| Expt. | N | Bladder | Ureter | Bladder | Ureter | Bladder | Ureter | Bladder | Ureter |
| 1 | 8 | 42·5±1·5 | 43·2±0·9 | 7·0±0·1 | 7·4±0·6 | 0·185±0·012 | 0·151±0·012 | 0·356±0·020 | 0·350±0·012 |
| 2 | 8 | 27·2±1·3 | 28·1±2·0 | 5·0±0·1 | 5·2±0·4 | 0·260±0·073 | 0·175±0·026 | 0·468±0·058 | 0·240±0·026 |
| 3 | 7 | 32·0±0·9 | 32·5±0·9 | 7·1±0·2 | 7·0±0·1 | 0·110±0·024 | 0·090±0·013 | 0·121±0·012 | 0·097±0·012 |
| 4 | 8 | 26·2±1·2 | 26·1±1·2 | 6·2±0·6 | 6·1±0·4 | 0·197±0·088 | 0·100±0·026 | 0·220±0·068 | 0·132±0·053 |
| 5 | 8 | 33·4±1·3 | 33·6±1·5 | 6·7±0·2 | 6·8±0·3 | 0·163±0·060 | 0·140±0·022 | 0·220±0·073 | 0·186±0·042 |
| 6 | 7 | 28·6±1·6 | 28·9±0·8 | 5·9±0·4 | 6·0±0·3 | 0·225±0·078 | 0·187±0·034 | 0·261±0·046 | 0·210±0·031 |

The figures represent the average and mean variation of N observations in each experiment.
* Expressed in Fishman Units where one unit releases 1μg. phenolphthalein from 0·5 mM-phenolphthalein β-D-glucuronide per hour in 0·1 M-sodium acetate-acetic acid buffer pH 4·5 at 37·5° C.

The individual enzyme titres in the samples of urine which had been in contact with the bladder epithelium were generally higher than the corresponding ureteric urine samples.

*The effect of length of time of contact of isotonic saline with the bladder wall on β-glucuronidase activity*

The second ureter was ligated and severed cephalad to the ligature. The bladder was washed out once with saline, and then further saline was introduced. At intervals, 2 ml. samples of saline were withdrawn and analysed for creatinine concentration and β-glucuronidase activity.

The results are given in Table II.

The β-glucuronidase in the saline increased with the length of time the saline remained in contact with the bladder epithelium.

*Histological distribution of β-glucuronidase*

The epithelium of both the ureter and the bladder was found to have a high concentration of β-glucuronidase. The sub-mucosal areas showed little activity

TABLE II.— *Urinary β-glucuronidase Activity in Bladders of Monkeys Isolated from the Kidneys, but Otherwise Intact*

| | Monkey No. 1 | | Monkey No. 2 | | Monkey No. 3 | |
|---|---|---|---|---|---|---|
| Time (minutes) | Creatinine optical density | β-glucuronidase Fishman units per ml. urine | Creatinine optical density | β-glucuronidase Fishman units per ml. urine | Creatinine optical density | β-glucuronidase Fishman units per ml. urine |
| 5 | — | — | — | — | 0·40 | 0·15 |
| 10 | 0·20 | 0·27 | 0·15 | 0·37 | 0·40 | 0·17 |
| 20 | 0·10 | 0·40 | 0·20 | 0·44 | 0·42 | 0·19 |
| 30 | 0·20 | 0·47 | 0·15 | 0·48 | 0·50 | 0·24 |
| 60 | 0·10 | 0·73 | 0·15 | 0·62 | 0·40 | 0·27 |

except in the walls of the blood vessels. The muscular layers revealed a marked activity.

### DISCUSSION

Histochemical preparations demonstrated a high activity of β-glucuronidase in the epithelium of the bladder and also of the ureter which is similar in structure, but offers a much smaller surface area to the urine (Fig. 1 and 2).

The increase in enzyme activity with the length of time that the saline was in contact with the bladder epithelium can be explained either as leaching from the surface of adsorbed β-glucuronidase originating from the kidney or as a change in volume of the saline. The creatinine concentration of the saline did not alter, and therefore there was no volume change. The consistently higher enzyme titre in the bladder urine as compared with the ureteric urine supports the thesis that the urinary β-glucuronidase consists of two components : one, the larger, originating from the kidney and a second, smaller fraction derived from the epithelium of the bladder and, to a smaller extent, the ureters.

Recently, Kerr, Barkin, D'Aloisio and Menezyk (1963) have been able to study an intact bladder in a man whose ureters were surgically re-directed into the colon. They found that the introduction of solutions of increasing specific gravity into the bladder lumen was followed by an increasing β-glucuronidase titre after 2 or 4 hours incubation in the bladder. pH had little or no effect on the phenomenon.

Whereas the present experiments were designed to eliminate the possibility of passive diffusion across the cellular membranes due to osmotic gradients by using isotonic saline, these workers simulated the osmotic effects of various concentrations of urine, and therefore it is possible that the higher titres obtained in the more concentrated solutions reflect not a secretion as they suggest but a movement along an osmotic gradient from the cell contents to the urine.

The amount of cellular debris found in our experiments was very small, and often was not detectable. Moreoever, the solution in contact with the bladder wall in the second experiment was isotonic saline which prevented osmotic changes from taking place in the epithelial cells.

The bladder fraction probably does not arise from the lysis of cells sloughed off by the urine, but there is no reason to doubt that injury to the epithelium would cause an increase by the damaged cellular contents disintegrating which would release the lysosomes into the urine.

Riotton and Fishman (1953) observed that, after the administration of testosterone propionate to male mice, urinary β-glucuronidase activity began to increase on the same day as the renal tissue β-glucuronidase and not before. Their inference was that the increased titre arose by the enzyme finding " its way from the kidney tubules directly into the urine without delay and suggests a phenomenon localised to the genito-urinary apparatus ".

Evidence that the urinary enzyme concentration is not related to the plasma enzyme levels was given by Goldbarg, Pineda, Banks and Rutenburg (1959) who said that conditions which raised the plasma levels did not necessarily raise the urinary levels. If the urinary enzyme is derived from the glomerular filtrate, then an indication of a link between plasma and urinary levels should have been obtained. This would therefore suggest that part, at least, of the enzyme titre is derived from active secretion by the kidney tubules, a process which is independent of the plasma concentration.

A comparison of correlation coefficients between β-glucuronidase levels in the ureteric urines and PAH clearances on the one hand and the enzyme levels and creatinine clearances on the other, was inconclusive.

In the six experiments, three revealed correlation coefficients of 0·748 or higher ($P \leq 0{\cdot}05$) between the enzyme and PAH clearances but only one when the correlation coefficients between the enzyme and creatinine clearances were considered. This was one in which the PAH enzyme correlation coefficient was higher.

The number of samples in each experiment was small ($N = 7$ or 8). Therefore, the individual errors had marked effects. At least 40 samples would have been necessary to obtain statistically significant results ($P \leq 0{\cdot}05$).

Creatinine clearances measure the filtration rate whilst PAH clearances are indicative of the renal plasma flow by virtue of it being both filtered at the glomeruli and actively secreted by the tubular epithelium. The closer correlation between PAH clearances and β-glucuronidase activity suggests that some of the urinary enzyme may arise by tubular secretion which agrees with the findings of Riotton and Fishman (1953) and Goldbarg *et al.* (1959).

Urinary β-glucuronidase can therefore be assumed to arise from the following sources in possible order of magnitude :

(1) Glomerular filtration
(2) Tubular secretion
(3) Bladder (and ureteric) epithelial secretion
(4) Cellular lysis following epithelial tissue damage.

### SUMMARY

1. Part of the β-glucuronidase present in normal urine originates from the bladder epithelium.

2. Renal tubular activity is responsible for part of the urinary β-glucuronidase originating from the kidney.

---

### EXPLANATION OF PLATE

FIG. 1.—Vervet monkey. T.S. ureter stained to show the distribution of β-glucuronidase activity (8-hydroxyquinoline glucuronide method).

FIG. 2.—Vervet monkey. T.S. urinary bladder stained to show the distribution of β-glucuronidase activity (8-hydroxyquinoline glucuronide method).

1

2

Fripp.

Financial assistance in the form of a Grant-in-Aid from the Rockefeller Foundation and a grant from the British Empire Cancer Campaign for Research is gratefully acknowledged.

The clearance determinations were kindly performed by Dr. M. A. Crawford.

### REFERENCES

Boyland, E., Gasson, J. E. and Williams, D. C.—(1957) *Brit. J. Cancer*, **11**, 120.

*Idem*, Wallace, D. M. and Williams, D. C. (1955) *Ibid.*, **9**, 62.

de Duve, C., Pressman, B. C., Gianetto, R., Wattiaux, R. and Appelmans, F.—(1955) *Biochem. J.*, **60**, 604.

Fishman, W. H.—(1955) *Advanc. Enzymol.*, **16**, 361.

*Idem* and Baker, J. R.—(1956) *Cancer*, **12**, 240.

*Idem*, Springer, Belle and Brunetti, R.—(1948) *J. biol. Chem.*, **173**, 449.

Fripp, P. J.—(1961) *Ann. trop. Med. Parasit.*, **55**, 328.—(1963) *Biochem. J.*, **89**, 74P.

Goldbarg, J. A., Pineda, E. P., Banks, B. M. and Rutenburg, A. M.—(1959) *Gastroenterology*, **36**, 193.

Kerr, W. K., Barkin, M., D'Aloisio, J. and Menezyk, Z.—(1963) *Cancer*, **16**, 633.

King, E. J. and Wootton, I. D. P.—(1956) ' Micro-analysis in medical biochemistry '. 3rd edition. London (J. & A. Churchill, Ltd).

Levvy, G. A.—(1952) *Biochem. J.*, **52**, 464.—(1956) *Vitam. & Horm.*, **14**, 267.

*Idem*, and Marsh, C. A. (1959) *Advanc. Carbohyd. Chem.*, **14**, 381.

Riotton, G. and Fishman, W. H.—(1953) *Endocrinology*, **52**, 692.

Talalay, P., Fishman, W. H. and Huggins, C.—(1946) *J. biol. Chem.*, **166**, 757.